# Current Research on Suicide in Older Adults

**DOI:** 10.1093/geroni/igab046.2018

**Published:** 2021-12-17

**Authors:** Montgomery Owsiany, Yeates Conwell

## Abstract

Rates of suicide are elevated in middle- and late-life, yet studies focusing on suicidal ideation and behavior in older adults are limited compared to research in younger adults. The studies included in the present symposium offer valuable findings on suicide in older adults across the span of late-life. Owsiany et al. focus on age differences between older and younger adults in the association between anxiety symptoms and suicide risk. In Heisel et al., an online intervention is assessed for improving the outcomes of psychological well-being and suicide risk in older adult men who are transitioning into retirement during the COVID-19 pandemic. Crnek-Georgeson and Wilson reviewed the link between retirement patterns and psychological effects, including suicidal behaviors, among older adults. Additionally, this review includes recommendations for policy makers and employers in an effort to assist older adults with the transition into retirement. Utilizing baseline data from the Helping Older Adults Engage study, Fenstermacher et al. research the association between volunteering and suicidal ideation in a predominantly lonely older adult sample across the span of late-life. Together, these studies provide foundation for future research on suicide in late-life to build upon. Future studies should continue to focus on risk and protective factors for suicide in older adults and aim to improve screening and intervention for suicidal thoughts and behaviors in this population. Yeates Conwell, M.D., Director of Geriatric Psychiatry and Co-Director of the Center for the Study and Prevention of Suicide at the University of Rochester Medical Center, will serve as discussant.

